# Accumulating candidate genes for broad-spectrum resistance to rice blast in a drought-tolerant rice cultivar

**DOI:** 10.1038/s41598-021-00759-9

**Published:** 2021-11-02

**Authors:** Maria Gay C. Carrillo, Federico Martin, Mukund Variar, J. C. Bhatt, Alvaro L. Perez-Quintero, Hei Leung, Jan E. Leach, Casiana M. Vera Cruz

**Affiliations:** 1 International Rice Research Institute (IRRI), DAPO Box 7777, Metro Manila, Philippines; 2 Agricultural Biology, Colorado State University, 307 University Avenue, Fort Collins, CO 80523-1177 USA; 3 Central Rainfed Upland Rice Research Station, PO Box 48, Hazaribag, 825 301 India; 4 ICAR-Vivekananda Parvatiya Krishi Anusandhan Sansthan (VPKAS), Almora, Uttarakhand India

**Keywords:** Genetics, Plant sciences

## Abstract

Biotic stresses, including diseases, severely affect rice production, compromising producers’ ability to meet increasing global consumption. Understanding quantitative responses for resistance to diverse pathogens can guide development of reliable molecular markers, which, combined with advanced backcross populations, can accelerate the production of more resistant varieties. A candidate gene (CG) approach was used to accumulate different disease QTL from Moroberekan, a blast-resistant rice variety, into Vandana, a drought-tolerant variety. The advanced backcross progeny were evaluated for resistance to blast and tolerance to drought at five sites in India and the Philippines. Gene-based markers were designed to determine introgression of Moroberekan alleles for 11 CGs into the progeny. Six CGs, coding for chitinase, HSP90, oxalate oxidase, germin-like proteins, peroxidase and thaumatin-like protein, and 21 SSR markers were significantly associated with resistance to blast across screening sites. Multiple lines with different combinations, classes and numbers of CGs were associated with significant levels of race non-specific resistance to rice blast and sheath blight. Overall, the level of resistance effective in multiple locations was proportional to the number of CG alleles accumulated in advanced breeding lines. These disease resistant lines maintained tolerance to drought stress at the reproductive stage under blast disease pressure.

## Introduction

Rice (*Oryza sativa*) is grown on more than 147 million hectares worldwide, primarily in countries with high human population densities^1^. Mounting constraints to rice production, which include increasing global temperatures, decreasing land spaces for production, depleting water supplies, as well as other abiotic and biotic stresses threaten the security of food supply. Of the biotic stresses affecting rice production, diseases pose a major threat. Rice blast caused by the fungus *Magnaporthe oryzae* is a principal disease of rice due to its wide geographical distribution and its destructiveness under favorable conditions^2^. Breeding for resistant cultivars containing major resistance (*R*) genes has been a key means to managing blast infection with approximately 100 *R* genes available for use against multiple races of the pathogen^3–5^. Even with this large number of *R* genes, loss of resistance, largely due to pathogen adaptation, has been well documented^2,6,7^.

The development of cultivars with more stable forms of blast resistance is highly desirable, prompting the exploration of additional strategies beyond the accumulation of *R* genes against specific pathogens. Durable resistance is associated with polygenic partial/quantitative resistance which typically shows no race specificity^8–11^. The candidate gene approach, which is based on the principle that genes with known functions in the traits of interest may reside in major loci^9^, has been used to map known genes to QTL of multiple traits, including disease resistance, in various rice mapping populations^10–14^. These CGs included resistance gene analogs and a number of defense response genes that code for chitinase (CHI), oxalate oxidase (OXO), germin-like proteins (GLP), peroxidase (POX), phenylalanine ammonia lyase (PAL), superoxide dismutase, 14-3-3 proteins, and thaumatin-like protein (PR5), which are associated with QTL conferring resistance to fungal and bacterial pathogens of rice as well as resistance to brown plant hopper^15–22^. This frame map has been a useful reference for selecting CGs involved in both pathogen recognition and general plant defense, and has also been important for analysis of mapping populations to improve resistance to rice blast^10,13^.

Simultaneous detection of major genes and/or QTL for blast resistance and their transfer to elite backgrounds can accelerate the process of varietal development^23^. Using an advanced backcross method, Moroberekan, a traditional rice variety from the Ivory Coast, was employed in hybridization to improve blast resistance in a popular drought-tolerant upland rice variety Vandana from Eastern India^13^. Seven QTL were identified when BC_2_F_4_ and BC_3_F_4_ populations were analyzed: two QTLs from Vandana and four from Moroberekan conferred resistance to leaf blast, and one QTL from Moroberekan reduced panicle blast severity.

In this paper, we developed cultivars with QTL-based resistance to rice blast by pyramiding different combinations of CGs associated with rice blast QTL while simultaneously selecting progenies with good agronomic traits for cultivar release. Our strategy involved intermating previously produced BC_3_F_4_ families from Vandana/Moroberekan crosses that showed partial resistance to rice blast^13^ to produce lines with different combinations of CGs. The intermated progenies were screened for resistance to rice blast at locations with high levels of disease and pathogen-diversity in India (Almora, Hazaribag and Ambikapur) and the Philippines (Cavinti and the IRRI blast nursery). Progenies exhibiting a good level of resistance to rice blast as well as desirable morphological and agronomic traits were advanced to the next generation. The advanced lines containing multiple CGs exhibited effective broad-spectrum resistance to rice blast at multiple locations, showing the potential of pyramiding CGs for disease resistance.

## Results and discussion

### Disease resistance of F_4_ lines from intermated BC_3_F_4_ of Vandana/Moroberekan

**Figure 1:**
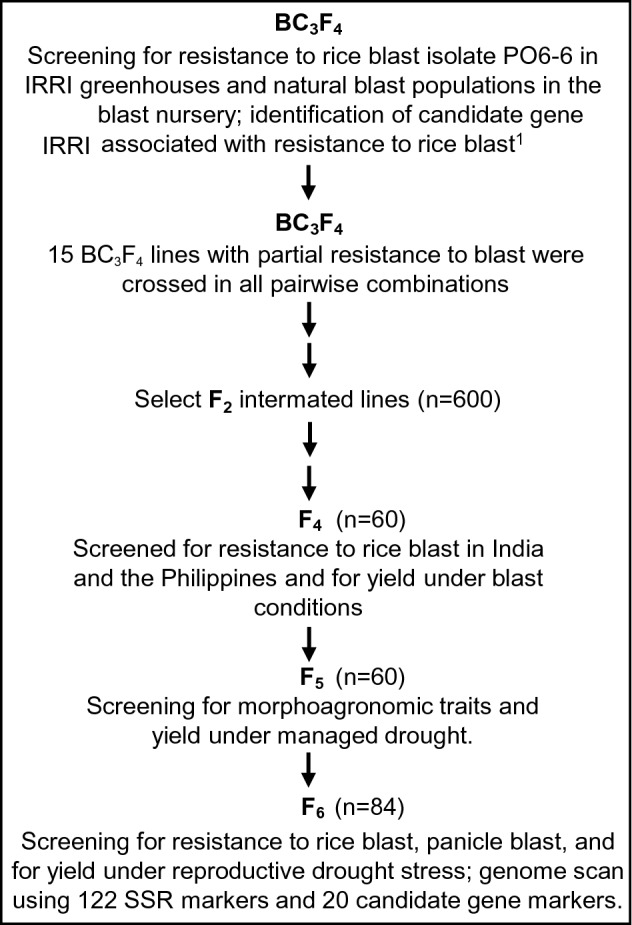
Breeding scheme describing the development of intermated lines from BC_3_F_4_ Vandana/Moroberekan families generated by Wu et al.^13^.

Moroberekan (IRTP 19187), a japonica cultivar with durable resistance to rice blast, was crossed to Vandana (IRTP 932), a popular drought-tolerant but blast-susceptible variety from India, and the population was advanced to BC_3_F_3_ and BC_3_F_4_^13^. Fifteen lines from the BC_3_F_4_ were crossed in all pairwise combinations and then selfed until F_6_ (Fig. 1). At the F_4_ stage, lines were screened for field resistance to blast at five sites in India and the Philippines. A comparison of the mean trait value for reaction to rice blast showed that Vandana was consistently more susceptible than Moroberekan across all sites, while intermated lines exhibited a range of responses to seedling blast, from resistant to highly susceptible (2.5–8.5, SES scale). Measurement of Diseased Leaf Area (DLA) 7 weeks after sowing confirmed that Vandana was highly susceptible to blast (65% DLA) compared to Moroberekan (1.5% DLA) in Almora. Among the intermated lines, variations in DLA were also observed with scores reaching up to 70% but with a mean value of just over 12% (Table 1). A wide variation in panicle blast (PB) severity was observed in Almora, with SES ranging from 3.0 to 8.3. While Moroberekan was moderately resistant to PB (SES = 3.15), Vandana was highly susceptible, scoring an average rating of 8.3. The response to PB was more pronounced in Hazaribag, where the PB scores ranged from 0 to 34.5.

**Table 1:** Trait mean values for Vandana, Moroberekan and F_4_ intermated BC_3_F_4_ lines screened for resistance to rice blast at multi-location trials in India and the Philippines.

Trait	Vandana	Moroberekan	Intermated F_4_ lines from BC_3_F_4_	
			Mean	Range
**India**				
a. Almora				
Lesion number	3.3	0.7	1.7	0.3–4.9
Seedling blast^1^	8.5	3.5	5.7	2.5–8.5
DLA^2^	65	1.5	12.2	1.0–70.0
Start of disease^3^	15	20	17.3	15–25
Panicle blast^1^	8.3	3.2	5.4	3.0–8.3
Plot yield^4^	5.0	–^5^	68.3	5.0–170.0
b. Hazaribag				
Leaf blast^1^	3	2	2.4	2.0–4.0
Panicle blast^4^	10.8	0	13.0	0.0–34.5
c. Ambikapur				
Leaf blast^1^	4	3	3.3	1.0–4.0
**Philippines**				
a. IRRI				
Seedling blast^1^	5	1	2.6	1.0–6.0
b. Cavinti				
Leafblast^1^	7	2	2.5	1.0–7.0

^1^Measure using SES (IRRI, 1996) Scale 0–9.

^2^*DLA* Diseased leaf area (%) at seven weeks after sowing.

^3^Days after sowing.

^4^Plot yield (grams per plot) under field rice blast infection.

^5^Data not available.

^6^Panicle blast severity measured as ${\text{Panicle blast severity }} = \frac{{\left( {{1}0 \times {\text{N1}}} \right) + \left( {{2}0 \times {\text{N3}}} \right) + \left( {{4}0 \times {\text{N5}}} \right) + \left( {{7}0 \times {\text{N7}}} \right) + \left( {{1}00 \times {\text{N9}}} \right)}}{{{\text{Total }}\# {\text{ of panicles observed}}}},$ where N1–N9 are the # of panicles with score 1–9.

Agronomic performance under field blast condition was only recorded in Almora. Vandana yielded poorly (5 g per plot) under heavy field blast, while intermated lines yielded from 5 to 170 g per plot (Table 1). Most trait means for blast resistance were more similar to Moroberekan than to Vandana. The progenies were moderately to completely resistant to blast and yielded better under blast conditions than either of the parents (Table 1). In Almora, DLA and yield under blast (r =  − 0.60***) and panicle blast severity and yield under blast (r =  − 0.81***) were negatively correlated, while the start of disease symptoms is positively correlated to yield under blast (r = 0.62***) (Table S5). Taken together, these data show that although the intermated lines are phenotypically most similar to Vandana, they were more resistant to blast and yielded better under blast conditions than Vandana.

The disease response of intermated lines was equally correlated across the multiple locations. DLA at Almora showed positive correlation with lesion number (LN) scores in Hazaribag (r = 0.54**—Fig. S1) and LN in Hazaribag and Ambikapur were also similarly correlated (r = 0.54**—Fig. S2). Additionally, seedling blast (SB) measurements performed at the IRRI blast nursery were well correlated with panicle blast in Almora (p < 0.01), as well as seedling blast in Cavinti (p < 0.0001) (Table S5). Blast races at these locations vary, as shown by a coordinated monitoring program of the lineages of *M. oryzae* and virulence on a set of international differential varieties in the Philippines and in India^24–26^. Thus, the correlation in disease reactions of the BC_3_F_4_ intermated lines at multiple locations, i.e., their similar behavior, suggests that quantitative resistance exhibited by these materials is race-nonspecific.

### Molecular analyses of intermated Vandana/Moroberekan lines

Cluster analysis of BC_3_F_4_ families using genotypic data showed higher similarity to the Vandana background (> 85%) relative to Moroberekan^13^. To better understand the contribution of accumulated CGs on the intermated lines under this genetic background, we utilized a combination of CGs specific markers, CGs associated SSR markers, and SSR markers randomly dispersed throughout the rice genome. The identified CGs included genes associated with disease resistance QTL in rice as well as genes induced by fungal or bacterial pathogens in rice or other species (Table S2). Twenty PCR primer pairs (Table S3) which detected polymorphisms in or near CGs and 31 SSR markers (Table S4) which co-localized with polymorphic CGs were used to identify CGs and introgressed regions from parental lines. In some instances, the SSR markers were more polymorphic than the CG-based PCR markers developed in this study (data not shown). An additional 122 polymorphic SSR markers were used to conduct genome-wide analysis (Table S6, Fig. S3).

Selected BC_3_F_4_ parents contained CGs in different number and combinations^13^. Line V4M-14-1-B, the common parent for all crosses (Table S1), contained Moroberekan alleles for the CGs that code for chitinase, oxalate oxidase, HSP90, thaumatin-like protein, peroxidase and probenazole-induced protein. V4M-5-3-B, V4M-6-1-B and V4M-82-2-B contained the Moroberekan allele for GLP. In addition, V4M-82-2-B also contained oxalate oxidase and DHAP from Moroberekan while V4M-6-1-B carried DHAP. CGs found in the parental lines were detected in various combinations in the selected progenies; several lines with no CG alleles from the Moroberekan parent were also identified.

**Figure 2:**
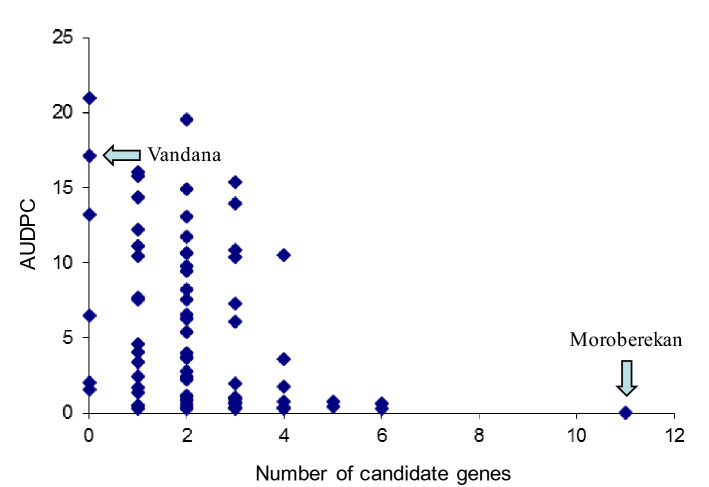
Performance of F_4_ intermated lines carrying different number of candidate genes measured by area under the disease progressive curve (AUDPC) in Almora, India.

**Figure 3:**
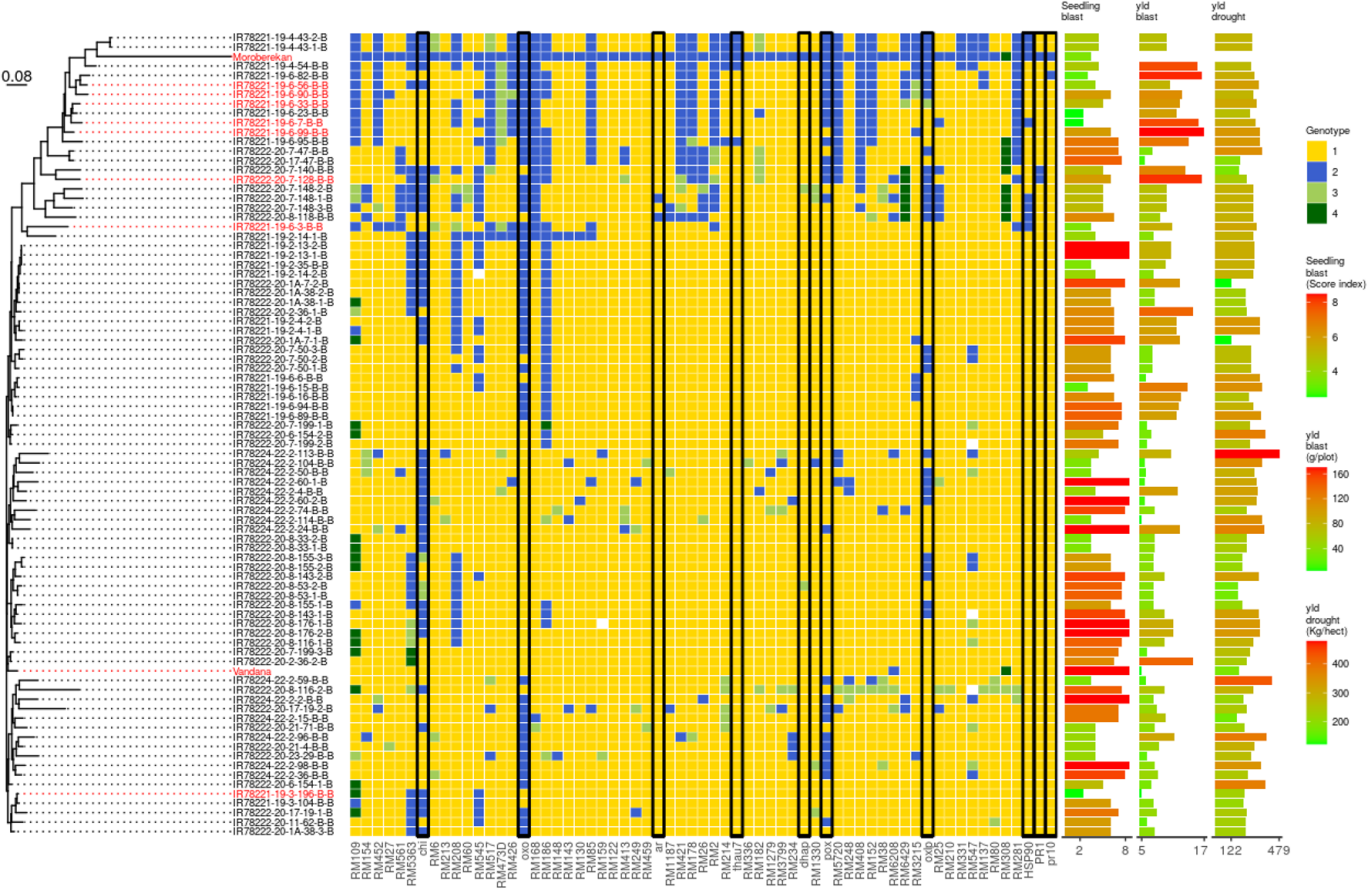
Neighbor-joining tree generated by calculating Gower distances between genotypes of F_6_ intermated lines based on the similarity to the population’s parents (left panel). Genotyping of intermated lines using 60 SSR markers spanning five chromosomes (see Fig. S3 for reference) and ten CG markers (black box highlight) shows greater similarity to Vandana (1—yellow) than Moroberekan (2—blue); some regions were heterozygous (3—light green), while others did not fit the above classes (4—dark green) (center panel). Lines accumulating Moroberekan regions performed better under seedling blast conditions in Almora (right panel). The same lines showed higher yields under heavy blast conditions. Performance under drought at IRRI is consistent with the parent Vandana. Chi (Chitinase), OXO (Oxalate Oxidase), ar (Aldose reductase), thau7 (Thaumatin like), pox (Peroxidase), oxlp (Germin-like protein), HSP90 (Heat shock protein 90), PR1 and PR10 (Pathogenesis-related genes).

**Figure 4:**
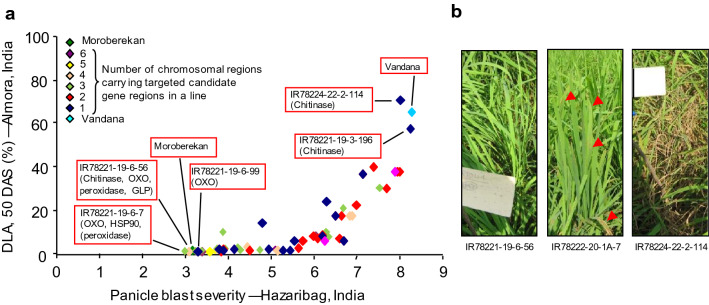
(**a**) Performance of F_6_ intermated lines with different number and candidate gene combinations (highlighted in red boxes) in Almora and Hazaribag, India. *DLA* disease leaf area, *DAS* days after sowing. (**b**) Leaf blast field phenotypes in Almora. Left: IR78221-19-6-56 (R); center: IR78222-20-1A-7 (S); right: IR78224-22-2-114 (HS). (R) Resistant; (S) Susceptible; (HS) Highly susceptible. Red arrowhead = blast lesions.

Analysis of disease progress curves of F_4_ intermated lines demonstrated that higher accumulation of CGs from Moroberekan contributed to a decrease in blast susceptibility in field experiments in Almora (Fig. 2). Interestingly, lines carrying five and six CGs were comparable to the monogenic lines carrying *Piz* and *Pi2*, *R* genes that are effective at all three locations^26^. Analysis of CGs accumulation on intermated F_6_ progeny showed a similar trend. Genotyping data from 60 SSR markers covering five chromosomes enriched in CGs were used to generate a neighbor-joining tree calculating Gower distances between the genotypes of F_6_ intermated lines (Fig. 3). The analysis verified the preponderance of the Vandana genotype and showed strong clustering of lines that had accumulated Moroberekan markers, including CG markers as well as CG-linked SSR markers (Fig. 3). Phenotypically, most F6 intermated lines with accumulated Moroberekan regions showed increased resistance to rice blast across screening sites in Almora and Hazaribag, as well as better yield under blast infection in Almora (Figs. 3, 4, Table 1). Genome-wide comparison among lines containing different combinations of CGs showed no significant influence from additional Moroberekan genomic regions (Fig. S4). Overall, while the monogenic lines generally exhibited an ‘all or nothing effect’ with high or low disease indices, the introgressed population had a range of disease intensities that declined progressively with the addition of each CG.

### Association of candidate genes with QR

Six CGs, coding for chitinase, HSP90, OXO, GLP, PR5, and POX, were correlated with blast resistance across all screening sites in India and the Philippines (Table 2). The exception was Hazaribag, where no measurable contribution to resistance was observed; Hazaribag typically has low blast pressure, particularly relative to Almora, so this result was not unexpected. Of the evaluated CGs, the genes coding for aldose reductase, OXO, GLP and POX are involved in oxidative stress, while chitinase is an enzyme that inhibits fungal growth by degrading chitin found in fungal cell walls^27–29^. HSP90 is an important chaperone protein found in many plant species and is involved in protection against multiple biotic as well as abiotic stresses^30^.

**Table 2:** Phenotypic contribution (percentages) of candidate gene allele to rice blast disease resistance in Vandana, Moroberekan and intermated F_6_ lines screened for resistance to rice blast at multi-location trials in India and the Philippines.

Gene	Marker	Trait^1^										
		India									*Philippines*	
		Almora						Hazaribag		Ambikapur	IRRI	*Cavinti*
		LN	SB	DLA	SD	PB	YB	LB	PB	LB	SB	*SB*
Chitinase	RM6379	7.19^b^	4.54^a^	3.84^a^	–	4.23^a^	–	–	–	4.31^a^	–	*–*
HSP90	HSP90	–	8.07^a^	4.82^a^	–	5.14^b^	–	–	–	–	–	*–*
OXO	*OsOxO4* UP	12.84^c^	14.31^c^	13.21^c^	5.70^a^	15.40^c^	12.28^c^	–	–	–	9.52^b^	*8.44*^*b*^
GLP	RM6429	8.77^a^	–	–	–	–	–	–	–	–	–	*–*
	RM3215	8.77^a^	–	–	10.29^b^	12.59^b^	11.53^b^	–	–	13.33^c^	8.28^a^	*6.97*^*a*^
	RM331	–	4.20^a^	–	–	–	–	–	–	–	–	*–*
PR5	RM214	5.01^a^	–	–	–	6.01^a^	4.80^a^	–	–	–	5.36^a^	*4.52*^*a*^
POX	RM5720	7.45^a^	6.80^a^	7.77^b^	–	13.45^b^	14.52^c^	–	–	4.63^a^	8.85^a^	8.06^a^

*LN* lesion number, *SB* seedling blast, *DLA* diseased leaf area, *SD* start of disease (days after sowing), *PB* panicle blast, *YB* yield under blast condition, *LB* leaf blast.

^1^Significance level of correlation is: ^a^p < 0.05; ^b^p < 0.01; ^c^p < 0.001; (–) not significant.

All CGs, to a certain degree, contributed to resistance in one or more locations (Table 2). Of the CGs evaluated, only the OXO allele detected by OXO-associated markers contributed significantly (p < 0.001) to all six blast resistance parameters measured in Almora. In the Philippines, OXO contributed significantly to seedling blast resistance in Cavinti (p < 0.01) and the IRRI blast nursery (p < 0.01). Interestingly, not all of the selected markers associated with the CG resistance allele detected the same levels of contribution to disease resistance. For example, of the two OXO gene-specific markers tested that were polymorphic between Vandana and Moroberekan, only *OsOXO4* UP marker was significantly associated with resistance conferred by *OsOXO4*. This result correlates with our previous study on the expression of *OsOXO4* during resistance to rice blast, and supports association with a missing 26-bp promoter region in the Vandana allele and selected blast-susceptible progeny^31^. The association to a phenotype and contribution level for each marker will depend on multiple factors including the polymorphism detected by the marker, the evolutionary history of the polymorphism in a population, and the quantitative nature of the trait. A marker detecting a causative polymorphism may show different association to a phenotype than a marker detecting a polymorphism linked to the causative one^32^. In addition, the quantitative nature of a trait will vary depending on the genetic background and environmental conditions tested leading to the fluctuating contribution by different elements associated with the trait.

Other CGs also showed significant correlation with blast resistance. POX showed association to resistance against DLA, decreased PB and yield under blast in Almora, while GLP exhibited significant association with start of disease, panicle blast and yield under blast as well as LB (Table 2). In addition, GLP showed high association with leaf blast infections in Ambikapur. Single gene analysis of variance (ANOVA) also correlated POX with yield under blast in Almora (p < 0.001); both POX and OXO appear to be important for panicle blast resistance at this location. In addition, while both OXO and PR5 are significantly associated with seedling blast resistance at the IRRI blast nursery, only PR5 is associated with panicle blast resistance in Cavinti. A two-gene ANOVA (p < 0.0001) revealed association of oxalate oxidase/thaumatin with yield under drought conditions and with seedling blast resistance at IRRI. Moreover, association between HSP90/thaumatin with seedling blast resistance was noted at IRRI nursery, and thaumatin/oxalate oxidase with panicle blast resistance in Cavinti.

The associations found using PCR markers for the CGs were confirmed using 26 SSR markers that are correlated with resistance to rice blast across screening sites in India and the Philippines (Table S7). These include SSR markers which co-localize with CGs. For example, SSR markers which co-localized with OXO (RM168 and RM426), POX (RM5720), and GLP (RM3215, RM25, and RM331) were highly correlated with resistance to blast across screening sites. OXO marker RM168 was previously identified as significantly associated with blast lesion number in Vandana/Moroberekan, as well as other rice varieties^13,33^. Moreover, comparative mapping of these SSRs using cMAP revealed that they co-localized with previously reported QTLs involved in resistance to bacterial blight, rice blast, sheath blight and/or brown planthopper (Table S8). No other SSR markers showed significant association with resistance to rice blast across the studied screening sites.

Overall, despite the different contribution of individual CGs or their combinations to disease response at the tested locations, results confirm that increasing numbers of CGs in a line correlated to lower susceptibility of F_6_ intermated progeny. Importantly, the use of CGs as markers was effective in determining the response of each line and demonstrated that accumulation of even a few QTL-based CGs can substantially reduce blast susceptibility.

### Accumulation of defense response genes is correlated to resistance

Screening of 60 F_4_ lines in blast endemic locations in India indicated that the disease progress curve declined progressively with addition of each CG (Fig. 2). Among these were several progenies carrying from 1 to 6 CG combinations which exhibited good morphoagronomic traits (Table S9). The performance of F_6_ intermated lines with different CG combinations in Almora and Hazaribag similarly demonstrated strong correlation between the level of resistance and the number of defense response genes in the intermated lines, and confirmed the results obtained with F_4_ generation. Lines carrying multiple CG alleles from Moroberekan showed lower DLA and lower panicle blast severity estimates. The leaf blast (Almora) and panicle blast (Hazaribag) data for these lines were comparable to the donor parent Moroberekan. In contrast, Vandana and lines with one or two CG alleles were more susceptible at both sites, but this depended on the CG present in the line (Figs. 3, 4). For example, the line IR78221-19-6-99-B-B which carried only OXO was less susceptible across screening sites. However, line IR78221-19-3-196-B-B, containing only the chitinase CG, was susceptible across screening sites. The presence of some single CGs conferred resistance while others, when present alone, did not, showing the differences in contribution of each gene. This was also apparent when the genes were combined in lines, that is, the resistance observed was not proportional to the number of CGs present. However, we did observe a significant reduction in leaf blast severity with increasing numbers of CGs in introgressed lines at different locations and years. This suggests that accumulation of CGs, conferring different mechanisms of resistance, contributes to resistance which is effective in multiple environments. Thus, our work confirms that selection for CGs is an effective means to improve quantitative disease resistance in rice^10,11,14^.

### Lines with accumulated CGs show broad spectrum resistance against sheath blight

**Figure 5:**
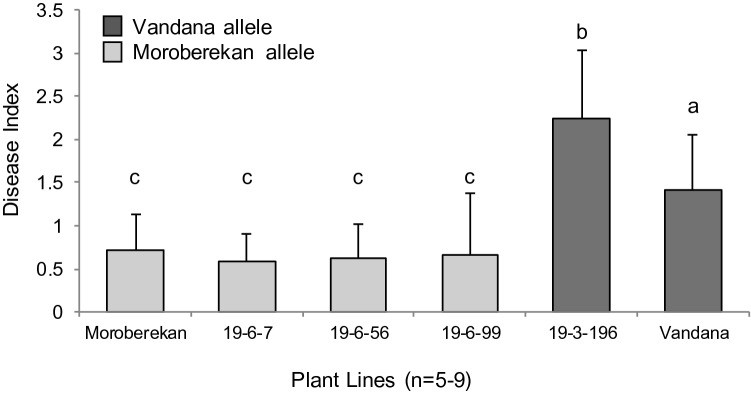
Performance of F_6_ intermated lines with different CG genes after inoculation with *R. solani* strain RM01401 in greenhouse tests. Introgressed lines with multiple CGs, particularly those with OXO, are more resistant than lines with few CGs. Line number (CG content): 19-6-7-B-B (OXO, HSP90, POX); 19-6-56-B-B (Chitinase, OXO, POX, GLP); 19-6-99-B-B (OXO); 19-3-196-B-B (Chitinase).

In the rice paddies, combinations of multiple biotic stresses are observed and these combinations contribute to higher yield losses. Thus, breeding for cultivars showing increased disease resistance against multiple pathogens provides immense advantages. Sheath blight, cause by *Rhizoctonia solani*, is also a devastating disease which affects rice production in many areas around the world. Given the strong response observed at the multiple tested locations against rice blast, and considering the combination of CGs obtained in the introgressed lines, we also tested several lines for resistance to sheath blight. Greenhouse tests performed with a virulent strain of *R. solani* (RM01401) demonstrated that lines with favorable CG combinations also showed decreased susceptibility to sheath blight (Fig. 5). Line IR78221-19-3-196 containing a single CG was more susceptible than even the susceptible parent line Vandana. Overall, these results are consistent with previous findings that accumulation of appropriate combinations of CGs can enhance resistance against pathogens other than just the rice blast pathogen.

### Combined blast resistance and drought tolerance

**Figure 6:**
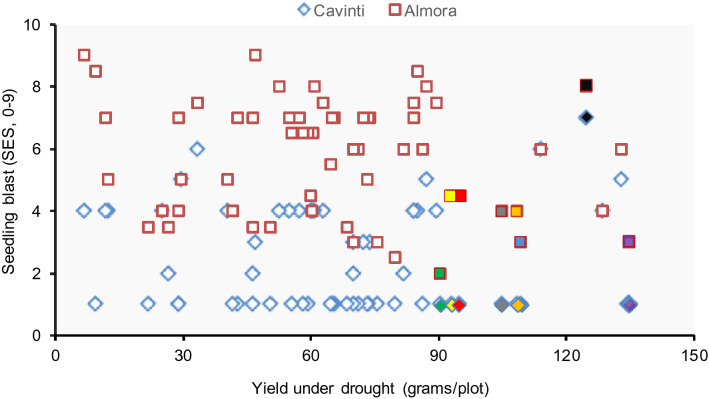
Yield during DS drought conditions at IRRI against seedling blast infection recorded at Cavinti and Almora of selected 60 F_5_ progenies from intermated BC_3_F_4_ lines. Highlighted in the graphic, filled data points correspond to lines (from left to right): IR78221-19-6-7-B-B (green); IR78221-19-6-3-B-B (yellow); IR78221-19-6-33-B-B (red); IR78222-20-7-128-B-B (gray); IR78221-19-6-90-B-B (orange); IR78221-19-6-99-B-B (blue); IR78221-19-6-56-B-B (purple); black (Vandana).

Because Vandana is a known drought tolerant cultivar from India, the Vandana/Moroberekan progenies were also evaluated for tolerance to drought at the reproductive stage. From all the tested lines, those which performed as well as or better than the Vandana parent during the reproductive stage (less than 10 cm rainfall for more than 30 days at rice reproductive stage) were selected. From 84 Vandana/Moroberekan F_6_ intermated lines tested, nine progeny were selected based on their resistance to rice blast across all screening sites (Table S9). These lines contained different CG allele combinations from Moroberekan, showed decreased DLA readings in Almora, and also were highly similar to Vandana (Table 3). Moreover, the selected lines also exhibited similar or better morphoagronomic traits than Vandana providing better harvest index and grain yield (Table 3). Thus, these lines are tolerant to drought at the reproductive stage while also showing enhanced resistance response against blast (Figs. 3, 6). Importantly, the introgression of CGs from Moroberekan into Vandana by the advanced backcross method did not compromise the drought tolerance provided by the latter. Thus, the advanced backcross method allowed for more rapid varietal development, combining both effective QTL-based disease resistance, a result of the simultaneous accumulation of CGs, and drought tolerance.

**Table 3:** Means of agronomic traits evaluated under drought condition of selected F_6_ Vandana/Morobekan intermated lines with resistance to field blast and the candidate genes detected in the genomes.

Line	Candidate gene	Harvest Index	Plant height (cm)	Grain yield (kg/ha)	Panicle weight (kg/ha)
Moroberekan	Donor	1.0	–^a^	181.6	525.14
IR78221-19-6-7-B-B	OXO, HSP90, POX	25.6	102.7	2765.0	2417.3
IR78221-19-6-33-B-B	OXO, POX, GLP	29.3	107.4	2117.0	2466.8
IR78221-19-4-54-B-B	CHI,OXO, THAU7, POX, GLP	16.17	124.4	1979.4	1893.3
IR78221-19-6-56-B-B	Chi, OXO, POX, GLP	19.9	109.4	2285.0	1823.0
IR78221-19-6-82-B-B	OXO, POX, GLP, PR10	19.5	113.5	2438.7	1884.3
IR78221-19-6-90-B-B	OXO, POX, GLP	21.5	115.5	2416.5	2496.7
IR78221-19-6-99-B-B	OXO	27.7	110.0	2941.7	2849.0
IR78222-20-7-148-2-B	Chi, OXO, HSP90, AR, POX, GLP	32.1	112.2	3506.2	2940.6
IR78221-19-3-196-B-B	Chi	29.3	107.4	2117.0	2467.0
Vandana	Donor	*27.1*	*113.8*	*2360.3*	1665.6

The lines were exposed to less than 10 cm rainfall for at least 30 days during the reproductive stage at IRRI, 2004 wet season.

^a^No data.

### Conclusions

To achieve effective levels of resistance that are broad-spectrum and durable, identification and deployment of both qualitative and quantitative resistances are desirable. However, it is difficult to accumulate quantitative resistance without knowledge of the underlying genetic control and corresponding mechanisms involved in resistance expression. In this study of QTL association mapping using advanced backcross populations, we show that relatively few chromosomal regions (5–10) which contain CGs exhibit effective broad-spectrum resistance without compromising desirable agronomic traits or drought tolerance. To contribute to the practicality of this approach, the genetic behavior of the QTL containing CGs in different genetic backgrounds needs to be investigated. This research will facilitate the selection of cultivars with broad-spectrum resistance against blast and other pathogen populations in diverse rice-growing environments.

## Materials and method

### Plant materials

Initial crosses of Vandana by Moroberekan were produced by Wu et al.^13^. Fifteen of the BC_3_F_4_ parental lines that showed partial resistance to rice blast and carried CG alleles from Moroberekan were selected and crossed in all pairwise combinations. Seeds of 10 F_2_ families were selected based on partial resistance of the BC_3_F_4_ parental lines to seedling blast and neck blast, association of the parental lines with positive alleles, and phenotypic similarity to Vandana. Resistant lines from selected intermated families were advanced until F_4_. At F_4_, the top 10% of the lines (60 out of > 600) derived from the progenies of BC_3_F_4_ crosses V4M-14-1-B × V4M-5-3-B, V4M-14-1-B × V4M-6-1-B, and V4M-14-1-B × V4M-82-2-B which exhibited acceptable agronomic traits were selected, and were designated as IR78221, IR78222, and IR78224, respectively (Table S1). Selected F_5_ plants were evaluated for morphoagronomic traits and drought tolerance. Sixteen F_5_ lines that were still segregating by morphology were advanced separately to F_6_, from which 84 lines were identified for molecular analyses. At the IRRI nursery, 24 BC_3_F_4_ intermated F_6_ lines selected for drought tolerance were analyzed for CGs from Moroberekan and for resistance to blast.

### In silico mapping of candidate genes in the rice genome

Eleven defense response CGs were analyzed in the intermated backcross lines of Vandana/Moroberekan (Table S2). CGs coding for 14-3-3 protein, aldose reductase, chitinase, heat shock protein 90 (HSP90), oxalate oxidase (OXO), germin-like proteins (GLP), PR1, PR5 (thaumatin-like proteins), peroxidases (POX), probenazole-induced protein, and putative 2-dehydro-3-deoxyphosphoheptonate aldolase (DHAP) were identified to be associated with quantitative resistance to fungal pathogens in rice mapping populations^10,12,13^. Rice orthologs of these CGs were mapped using BLASTn similarity searches against the rice genome database^34^.

### Molecular analysis of plant materials

SSR markers co-localizing with the CGs were identified using the Rice Genome Browser (http://www.gramene.org) of Gramene (Table S3), which maps the physical location of the CGs and displays molecular markers adjacent to the CG loci. PCR primers were designed based on the gene-coding region, the 3ʹ untranslated region, and the 1 kb upstream region of the CG rice sequences (Table S4). A survey of SSR polymorphism was conducted between Vandana and Moroberekan to identify polymorphic SSRs to scan the genome of the progenies. Polymorphic SSRs were used for genome-wide scans of the advanced backcross Vandana/Moroberekan progenies. Data generated from genome-wide scans informed statistical analyses for SSR markers contribution. Comparative mapping using the cMAP tool in Gramene was also used to identify QTL co-localizing with SSRs which were correlated with resistance to blast.

Two fresh leaves from ten 2-week-old plants were bulked, and DNA was extracted using the CTAB method^35^. DNA was amplified by PCR in a 50 μL volume. The final concentration of PCR reactions contained 100 ng of genomic DNA, 0.25 mM of each primer, 2 mM of each dNTP, 1.5 units *Taq* polymerase and 1× PCR buffer (New England Biolabs), under the following conditions: initial denaturation at 94 °C for 4 min; 35 cycles consisting of 1 min at 94 °C, 1 min at annealing temperature, and 2 min at 72 °C; and a final extension of 7 min at 72 °C. The PCR products from CG primers were resolved in 1.5% agarose gels while those from SSR markers were resolved in 2:1 2% metaphor:agarose gels. Gels were stained with ethidium bromide.

### Phenotyping of progenies

Plants were screened for resistance to rice blast in field locations in India (Almora, Hazaribag and Ambikapur) and in the Philippines (Cavinti and the IRRI blast nursery at Los Baños). Fifty seeds per line were sown per 1 m row; this was replicated three times in a randomized complete block design. Border rows consisting of a mixture of susceptible varieties were planted around each plot to enhance the pathogen population. In Almora, India, plants were grown to maturity and yield data (grams per plot) were collected. Scoring of leaf blast and panicle blast was based on the Standard Evaluation System (SES; http://www.knowledgebank.irri.org/images/docs/rice-standard-evaluation-system.pdf) for rice^36^. The progenies were also characterized for morphoagronomic traits and drought tolerance (http://inger.irri.org/).

Greenhouse sheath blight screenings were performed on 14-day-old plants using the microchamber assay^37^ and *Rhizoctonia solani* isolate RM01401. Plants were incubated in microchambers for 14 days post inoculation and were scored for both disease index (DI; lesion height/plant height × 9) and visual index (0–9 scale).

### Data analysis

A matrix of pairwise distances between varieties was generated by calculating distances between strings of coded genotypes, using the daisy function from the cluster library in R^38^. A neighbor-joining tree was obtained by using the nj function from the package ‘ape’ on the distance matrix^39^. The phylogenetic tree, the genotype data and accompanying data were visualized using the ggtree package^40^.

Simple and multiple regression analyses were conducted using the General Linear Model (GLM) procedure in SAS (SAS Institute, 1989) for test of association between CG and SSR markers with phenotypes at 0.05 and 0.01 levels of significance. Two-way and three-way ANOVA (SAS) were used to determine the best CG combinations per site. The PROC CORR procedure was used to estimate the Pearson correlation coefficient. Single-marker regression was analyzed using QGene^41^.

### Declaration

All studies in the manuscript comply with relevant institutional, national and international guidelines and legislation.

## Supplementary Information

Supplementary Information.

## Data Availability

All data generated or analyzed during this study are included in this published article (and its Supplementary Information files).
