# Supplementary material for: Accumulating candidate genes for broad-spectrum resistance to rice blast in a drought-tolerant rice cultivar
Source: Sci Rep. 2021 Nov 2;11:21502. doi: 10.1038/s41598-021-00759-9 (PMC8563964; doi:10.1038/s41598-021-00759-9)
Supplement: Supplementary file 1 — Supplementary Information. [file 41598_2021_759_MOESM1_ESM.pdf]

Accumulating candidate genes for broad-spectrum resistance to rice blast in a drought-tolerant rice cultivar

Maria Gay C. Carrillo[1*], Federico Martin[2*], Mukund Variar[3], J. C. Bhatt[4], Alvaro Perez-Quintero[2], Hei Leung[1], Jan E. Leach[2], and Casiana M. Vera Cruz[1]

Author Affiliation:

[1]International Rice Research Institute (IRRI), DAPO Box 7777, Metro Manila, Philippines; [2]Agricultural Biology, Colorado State University, Fort Collins, CO 80523-1177 USA; [3]Central Rainfed Upland Rice Research Station, PO Box 48, Hazaribag 825 301, India; [4]ICAR-Vivekananda Parvatiya Krishi Anusandhan Sansthan (VPKAS,) Almora, Uttarakhand, India.

Co-Corresponding Authors:

Casiana M. Vera Cruz
International Rice Research Institute (IRRI)
DAPO Box 7777, Metro Manila, Philippines
Phone: +63 (2) 580-5600
FAX: +63 (2) 580-5699
E-mail: C.VeraCruz@irri.org

Jan E. Leach
Agricultural Biology,
307 University Avenue
Colorado State University,
Fort Collins, CO 80523-1177 USA
Phone: +1-970-491-2924
Email: Jan.Leach@colostate.edu

* These authors contributed equally to this manuscript.

Supplementary Materials

Table S1. List of intermated lines derived from BC_3F_4 progenies of Vandana x Moroberekan used in this study.

Table S2. Candidate genes associated with resistance to rice blast used in this study.

Table S3. SSR markers co-localizing with candidate genes that map to disease resistance QTL and showing polymorphism between Vandana and Moroberekan.

Table S4. Primer sequences for candidate genes used in this study.

Table S5. Correlation coefficients between measured parameters for resistance to blast across screening sites in India and the Philippines.

Table S6. List of simple sequence repeat (SSR) markers used for a genome scan of 114 intermated progenies of Vandana x Moroberekan used in the study.

Table S7. Correlation of SSR markers to trait means for Vandana, Moroberekan and intermated BC_3F_4 lines screened for resistance to rice blast at multi-location trials in India and the Philippines.

Table S8. SSR markers correlated with resistance to rice blast in India and Philippines that co-localize with known QTL for resistance to biotic stress.

Table S9. Field performance of Vandana, Moroberekan and selected intermated BC_3F_4 lines screened for resistance to rice blast at multi-location trials in India and the Philippines.

Figure S1. Correlation of disease leaf area at Almora and lesion number at Hazaribag, India on F_4 lines from intermated BC_3F_4 families. Measurements showed a significantly positive correlation (r= 0.54**, n=80 2005). LN = Lesion number, DLA = Disease leaf area.

Figure S2. Correlation of Lesion Number (LN) in Ambikapur and lesion number in Hazaribag, India on F_4 lines from intermated BC_3F_4 families. LN counts at both sites showed a significantly positive correlation (r= 0.546**, n=80 2005).

Figure S3. Location of SSR markers and candidate genes (green rectangles) considered in the study. OXLP = GLP.

Figure S4. Diagram representing data from genome-wide scan of parental lines and a selected subset of F_6 intermated lines. Moroberekan (1 - red), and Vandana (2 - blue) genomic regions are shown in three selected lines IR78221-19-6-33-B (3), IR78221-19-6-90-B (4), IR78222-20-7-148-2-B (5) all of which show Resistant phenotype. While most of the genomic background is similar to Vandana, some regions, including QTLs containing CGs, are from Moroberekan. Green regions represent heterozygosity.

Literature Cited in the Supplemental Figures and Tables in Carrillo et al.

Table S1. List of intermated[1] lines derived from BC_3F_4 progenies of Vandana x Moroberekan that were used for this study.

Code	Line	Cross
VM-101	IR78221-19-2-4-2-B	V4M-14-1-B x V4M-5-3-B
VM-102	IR78221-19-2-4-1-B	V4M-14-1-B x V4M-5-3-B
VM-103	IR78221-19-2-13-1-B	V4M-14-1-B x V4M-5-3-B
VM-104	IR78221-19-2-13-2-B	V4M-14-1-B x V4M-5-3-B
VM-105	IR78221-19-2-14-1-B	V4M-14-1-B x V4M-5-3-B
VM-106	IR78221-19-2-14-2-B	V4M-14-1-B x V4M-5-3-B
VM-107	IR78221-19-2-35-B-B	V4M-14-1-B x V4M-5-3-B
VM-108	IR78221-19-3-104-B	V4M-14-1-B x V4M-5-3-B
VM-109	IR78221-19-3-196-B	V4M-14-1-B x V4M-5-3-B
VM-110	IR78221-19-3-222-B	V4M-14-1-B x V4M-5-3-B
VM-111	IR78221-19-6-6-B	V4M-14-1-B x V4M-5-3-B
VM-112	IR78221-19-6-15-B	V4M-14-1-B x V4M-5-3-B
VM-113	IR78221-19-6-16-B	V4M-14-1-B x V4M-5-3-B
VM-114	IR78221-19-6-89-B	V4M-14-1-B x V4M-5-3-B
VM-115	IR78221-19-6-94-B	V4M-14-1-B x V4M-5-3-B
VM-116	IR78221-19-6-99-B	V4M-14-1-B x V4M-5-3-B
VM-167	IR78221-19-4-43-1-B	V4M-14-1-B x V4M-5-3-B
VM-168	IR78221-19-4-43-2-B	V4M-14-1-B x V4M-5-3-B
VM-169	IR78221-19-4-54-B	V4M-14-1-B x V4M-5-3-B
VM-170	IR78221-19-6-3-B	V4M-14-1-B x V4M-5-3-B
VM-171	IR78221-19-6-7-B	V4M-14-1-B x V4M-5-3-B
VM-172	IR78221-19-6-23-B	V4M-14-1-B x V4M-5-3-B
VM-173	IR78221-19-6-33-B	V4M-14-1-B x V4M-5-3-B
VM-174	IR78221-19-6-56-B	V4M-14-1-B x V4M-5-3-B
VM-175	IR78221-19-6-82-B	V4M-14-1-B x V4M-5-3-B
VM-176	IR78221-19-6-90-B	V4M-14-1-B x V4M-5-3-B
VM-177	IR78221-19-6-95-B	V4M-14-1-B x V4M-5-3-B
VM-126	IR78222-20-7-47-B	V4M-14-1-B x V4M-6-1-B
VM-127	IR78222-20-7-50-1-B	V4M-14-1-B x V4M-6-1-B
VM-128	IR78222-20-7-50-2-B	V4M-14-1-B x V4M-6-1-B
VM-129	IR78222-20-7-50-3-B	V4M-14-1-B x V4M-6-1-B
VM-130	IR78222-20-7-199-1-B	V4M-14-1-B x V4M-6-1-B
VM-131	IR78222-20-7-199-2-B	V4M-14-1-B x V4M-6-1-B
VM-132	IR78222-20-7-199-3-B	V4M-14-1-B x V4M-6-1-B
VM-133	IR78222-20-8-33-1-B	V4M-14-1-B x V4M-6-1-B
VM-134	IR78222-20-8-33-2-B	V4M-14-1-B x V4M-6-1-B
VM-135	IR78222-20-8-53-1-B	V4M-14-1-B x V4M-6-1-B
VM-136	IR78222-20-8-53-2-B	V4M-14-1-B x V4M-6-1-B
VM-137	IR78222-20-8-116-1-B	V4M-14-1-B x V4M-6-1-B

[1]The BC_3F_3 and BC_3F_4 lines were intermated in all pairwise combinations and advanced to F_6. At F_6, 84 progenies were selected based on resistance to blast and morphoagronomic traits similar to Vandana.

Table S1 continued

Code	Line	Cross
VM-138	IR78222-20-8-116-2-B	V4M-14-1-B x V4M-6-1-B
VM-139	IR78222-20-8-143-1-B	V4M-14-1-B x V4M-6-1-B
VM-140	IR78222-20-8-143-2-B	V4M-14-1-B x V4M-6-1-B
VM-141	IR78222-20-8-155-1-B	V4M-14-1-B x V4M-6-1-B
VM-142	IR78222-20-8-155-2-B	V4M-14-1-B x V4M-6-1-B
VM-143	IR78222-20-8-155-3-B	V4M-14-1-B x V4M-6-1-B
VM-144	IR78222-20-8-176-1-B	V4M-14-1-B x V4M-6-1-B
VM-145	IR78222-20-8-176-2-B	V4M-14-1-B x V4M-6-1-B
VM-146	IR78222-20-11-62-B	V4M-14-1-B x V4M-6-1-B
VM-147	IR78222-20-17-19-1-B	V4M-14-1-B x V4M-6-1-B
VM-148	IR78222-20-17-19-2-B	V4M-14-1-B x V4M-6-1-B
VM-149	IR78222-20-21-4-B	V4M-14-1-B x V4M-6-1-B
VM-150	IR78222-20-21-71-B	V4M-14-1-B x V4M-6-1-B
VM-117	IR78222-20-1A-7-1-B	V4M-14-1-B x V4M-6-1-B
VM-118	IR78222-20-1A-7-2-B	V4M-14-1-B x V4M-6-1-B
VM-119	IR78222-20-1A-38-1-B	V4M-14-1-B x V4M-6-1-B
VM-120	IR78222-20-1A-38-2-B	V4M-14-1-B x V4M-6-1-B
VM-121	IR78222-20-1A-38-3-B	V4M-14-1-B x V4M-6-1-B
VM-122	IR78222-20-2-36-1-B	V4M-14-1-B x V4M-6-1-B
VM-123	IR78222-20-2-36-2-B	V4M-14-1-B x V4M-6-1-B
VM-124	IR78222-20-6-154-1-B	V4M-14-1-B x V4M-6-1-B
VM-125	IR78222-20-6-154-2-B	V4M-14-1-B x V4M-6-1-B
VM-178	IR78222-20-7-128-B	V4M-14-1-B x V4M-6-1-B
VM-179	IR78222-20-7-140-B	V4M-14-1-B x V4M-6-1-B
VM-180	IR78222-20-7-148-1-B	V4M-14-1-B x V4M-6-1-B
VM-181	IR78222-20-7-148-2-B	V4M-14-1-B x V4M-6-1-B
VM-182	IR78222-20-7-148-3-B	V4M-14-1-B x V4M-6-1-B
VM-183	IR78222-20-8-118-B	V4M-14-1-B x V4M-6-1-B
VM-184	IR78222-20-17-47-B	V4M-14-1-B x V4M-6-1-B
VM-151	IR78222-20-23-29-B	V4M-14-1-B x V4M-6-1-B
VM-152	IR78224-22-2-2-B	V4M-14-1-B x V4M-82-2-B
VM-153	IR78224-22-2-4-B	V4M-14-1-B x V4M-82-2-B
VM-154	IR78224-22-2-15-B	V4M-14-1-B x V4M-82-2-B
VM-155	IR78224-22-2-24-B	V4M-14-1-B x V4M-82-2-B
VM-156	IR78224-22-2-36-B	V4M-14-1-B x V4M-82-2-B
VM-157	IR78224-22-2-50-B	V4M-14-1-B x V4M-82-2-B
VM-158	IR78224-22-2-59-B	V4M-14-1-B x V4M-82-2-B
VM-159	IR78224-22-2-60-1-B	V4M-14-1-B x V4M-82-2-B
VM-160	IR78224-22-2-60-2-B	V4M-14-1-B x V4M-82-2-B
VM-161	IR78224-22-2-74-B	V4M-14-1-B x V4M-82-2-B
VM-162	IR78224-22-2-96-B	V4M-14-1-B x V4M-82-2-B
VM-163	IR78224-22-2-98-B	V4M-14-1-B x V4M-82-2-B
VM-164	IR78224-22-2-104-B	V4M-14-1-B x V4M-82-2-B
VM-165	IR78224-22-2-113-B	V4M-14-1-B x V4M-82-2-B

VM-166	IR78224-22-2-114-B	V4M-14-1-B x V4M-82-2-B

Table S2 Candidate genes associated with resistance to rice blast used in this study

Gene	Rice loci	Chr	Bioprocess	Functional evidence
14-3-3	LOC_Os02g36974	2	Primary metabolism/ cellular signal transduction	Disease resistance QTL [1]; Induced gene in rice-*M. oryzae* and rice-*X. oryzae* interactions [2].
Aldose reductase	LOC_Os05g39690	5	Stress response	Disease resistance QTL [1]; Associated with stress tolerance in mature seeds of rice [3]
Chitinase	LOC_Os03g04060	2	Chitin degradation	Disease resistance QTL[1]; Defense response by degrading fungal chitin [4]
Heat shock protein 90 (HSP90)	LOC_Os06g50300	6	Stress response	Pathogen-induced defense gene and chaperoning role in R-gene mediate resistance [5,6]
Oxalate oxidase (OXO)	LOC_Os03g48750	3	Oxidative burst, signaling, structure	Gene cluster as a disease resistance QTL [7-10]
	LOC_Os03g48760	3		
	LOC_Os03g48770	3		
	LOC_Os03g48780	3		
Germin-like proteins (GLP)	LOC_Os08g08920	8	Strengthening of cell wall during papilla formation in barley-powdery mildew interaction	Gene cluster as a disease resistance QTL [7-9]; Induced expression in rice-*M. oryzae* interaction [11,12].
	LOC_Os08g08960	8		
	LOC_Os08g08970	8		
	LOC_Os08g08980	8		
	LOC_Os08g08990	8		
	LOC_Os08g09000	8		
	LOC_Os08g09010	8		
	LOC_Os08g09020	8		
	LOC_Os08g09040	8		
	LOC_Os08g09060	8		
	LOC_Os08g09080	8		
	LOC_Os08g13440	8		
	LOC_Os08g35750	8		

Gene	Rice loci	Chr	Bioprocess	Functional evidence
Pathogenesis related protein 1 (PR1)	LOC_Os10g11500	10	Pathogenesis related protein	Gene cluster as a disease resistance QTL [1]; Involved in defense response against fungal and bacterial pathogens of plants [13]
Pathogenesis related protein 5 (thaumatin-like proteins)/PR5	LOC_Os06g47600	6	Pathogenesis related protein	Gene cluster as a disease resistance QTL [7]; [8], [9]; Antifungal activity by binding to beta-1,3-glucans [14,15]
	LOC_Os07g23470	7		
	LOC_Os12g43380	12		
Peroxidase	LOC_Os07g48020	7	Oxidative burst, signaling, structure	Gene cluster as a disease resistance QTL [1]. Involved in defense response to bacterial blight of rice [16]
Probenazole-induced protein	LOC_Os12g36880	12	Pathogenesis related protein	Induced expression by probenazole and by *M. oryzae* [17]
Putative 2-dehydro-3-deoxyphosphoheptonate aldolase (DHAP)	LOC_Os07g42960	7	Aromatic amino acid synthesis	Pathogen-induced defense gene in maize-*Puccinia sorghi* interaction (Bai et al., unpublished)

Table S3 - Primer sequences for candidate genes that were used in this study

Gene	Primer name	Chr	Forward Sequence (5'-3')	Reverse Sequence (5'-3')
14-3-3	14-3-3	2	GCTTTCCCGTGAGGAGAATG	GCCACCATGGTATTCTCAGC
Aldose reductase	AR	5	TGCTCACTCCGTTCAGACAG	CAGGTTCCGCAGCTGTCTAT
Chitinase	CHI	2	CGTCAAAGCCGGATGTCC	GCGGTAAACAACGGTACGATC
Heat shock protein 90 (HSP90)	HSP90	6	TGGTCACCCTTTGATTGACTC	AGATGAGCTCCCTCAGGAAA
Oxalate oxidase	OsOxo1	3	CAAACAAAGCCAGCTTGTCA	CCCATAACATGGGAGAAAGTT
	OsOxo2	3	CCAGCTCGTCACTGCTACTG	AGCGAAGAACGGTACGAAAA
	OsOxo3	3	CATAGCTCGATGTGCATGGT	GCCGACATTGAACTGGAAGT
	OsOxo4	3	GCCAGGTGCCATTTCATTAC	AGTACCCACCGGTGAACTTG
Oxalate oxidase (1 kb upstream)	OsOxO1 UP	3	AGCTTGTCACTGCGCTTCTT	GTGGCAATCTTGGAGGAGAA
	OsOxo2 UP	3	GCCTCTACGGGGTCTTGACT	AGCGAAGAACGGTACGAAAA
	OsOxo3 UP	3	GGACCCTCTCCAGGACTTCT	CACGGACCACCTTGGAAT
	OsOxo4 UP	3	ACCCTGACCCTCTCCAAGAC	CGGACCACCCTGGAGTAGTA
Germin-like protein	GLP	8	GCAATTAGAACGGTATGAGC	TCGACGACATTGAACTGGAGG
Pathogenesis related protein 1 (PR1)	PR1	10	TTACGCACCCAATTGTAGCA	TCCCAGAGTCACAAACATCG
Pathogenesis related protein 5 (PR5)	PR5_6	6	CCAAATTCCACGAATATCCAA	TACCTGAGGTCCTCCCATCA
	PR5_7	7	TCTTGGTTGCTTCCGTTCTT	CGCCTGAAGAGAAATGCAA
	PR5_12	12	TGGATTCACAAACCACGAAA	TTGTCTTCGGGGAACAGGTA
Peroxidase	POX	7	TGATCAGAAGCCATATGTAGC	ATCAGCTGAGCATCTCATATAAGG
Probenazole-induced protein	PR10	12	ATGCCCAGGTCTCAAATGTC	TCTCCGTCGAGTGTGACTTG
Putative 2-dehydro-3-deoxyphosphoheptonate aldolase (DHAP)	DHAP	7	GACCGTGTAAGTAATGGGTGGT	GGCTGAGCAGTCATAGAAGAGG

Table S4 SSR markers co-localizing with candidate genes that map to disease resistance QTL and showing polymorphism between Vandana and Moroberekan

**Candidate Gene**	**Chr**	**cM**	**SSR Marker[1]**
14-3-3	2	88.2	RM6056, RM5363
Chitinase	2	93.2	RM6379
Oxalate oxidase	3	122.8	RM426, RM504, RM168
Aldose Reductase	5	95.8	RM188, RM1187
PR5 (Thaumatin-like)	6	113.4	RM340, RM6811
HSP90	6	121.7	RM176, RM6563, RM1150
PR5 (Thaumatin-like)	7	50	RM214, RM542
Putative DHAP	7	78.9	RM478, RM234, RM1330
Peroxidase	7	115.5	RM5720
Germin-like protein	8	38.5	RM544, RM310, RM6208, RM547
Germin-like protein	8	48.8	RM6429, RM3215,
Germin-like protein	8	53.7	RM331, RM126
PR1b	10	15.2	RM216, RM5348, RM311
Probenazole-induced protein	12	94.7	RM1015

[1] SSRs were selected based on anchor markers from BAC clones with the candidate gene loci found in www.gramene.org. Primer sequences are available at http://143.48.220.116/microsat/index.html.

Table S5. Correlation coefficients between measured parameters for resistance to blast across screening sites in India and the Philippines

**SSR Marker**	**Trait[1,2]**										
	India									Philippines	
	Almora						Hazaribag		Ambikapur	IRRI	Cavinti
	LN	SB	DLA	SD	PB	YB	LB	PB	LB	SB	SB
**India**											
Almora											
LN											
SB	0.05										
DLA	0.77[d]	-0.16									
SD	-0.49[d]	0.02	-0.47[d]								
PB	0.78[d]	0.03	0.79[d]	-0.65[d]							
YB	-0.60[d]	-0.06	-0.60[d]	0.62[d]	-0.81[d]						
Hazaribag											
LB	0.11	-0.02	0.14	-0.11	0.15	-0.32[b]					
PB	-0.17	-0.12	-0.17	-0.08	-0.19	0.12	0.00				
Ambikapur											
LB	0.21	-0.04	0.20	-0.10	0.23	-0.25[a]	0.28[a]	-0.18			
**Philippines**											
IRRI											
SB	0.30[b]	0.20	0.20	-0.26	0.37[b]	-0.32[a]	0.13	-0.31[b]	0.28[a]		
Cavinti											
SB	0.30[b]	0.10	0.28	-0.27	0.41[c]	-0.35[b]	0.16	-0.29[a]	0.34[b]	0.95[d]	

[1] LN = lesion number; SB = seedling blast; DLA = diseased leaf area; SD = start of disease (days after sowing); PB = panicle blast; YB = yield under blast condition; LB = leaf blast

[2] Significance of correlation: a = $p < 0.05$; b = $p < 0.01$; c = $p < 0.001$; d = $p < 0.0001$

Table S6. List of simple sequence repeat (SSR) markers used for a genome scan of 114 intermated progenies of Vandana x Moroberekan that were used in the study.

Marker	Map	Chr	Position cM	Motif	Annealing Tm (°C)	Product Size (bp)	GenBank Accession	References
RM495	IRMI 2003	1	0.3	(CTG)7	55	159	AQ573939	[18]
RM84	CORNELL SSR 2001	1	26.2	(TCT)10	55	113	AF343869	[19]
RM220	CORNELL SSR 2001	1	28.4	(CT)17	55	127	AF344041	[19]
RM1	CORNELL SSR 2001	1	29.7	(AG)26	55	113	AF344003	[20]
RM581	IRMI 2003	1	49	(GA)12	55	143	AP000969	[18]
RM490	CORNELL SSR 2001	1	51	CT)13	55	101	AQ510019	[18]
RM24	CORNELL SSR 2001	1	78.4	(GA)29	55	192	AF343841	[19]
RM5	IRMI 2003	1	98.5	(GA)14	55	113	AF344007	[20]
RM246	CORNELL SSR 2001	1	115.2	(CT)20	55	116	AF344066	[19]
RM302	IRMI 2003	1	135.8	(GT)30(AT)8	55	156	AF344127	[21]
RM104	IRMI 2003	1	159	(GA)9	55	222		[22]
RM414	IRMI 2003	1	161.5	(TGCA)6	55	223	AQ158869	[18]
RM14	CORNELL SSR 2001	1	194.1	(GA)18	55	191	AF344015	
RM109	IRMI 2003	2	0	(AG)16	55	97	D15964	[21]
RM154	CORNELL SSR 2001	2	4.8	(GA)21	55	183	D39059	[22]
RM452	CORNELL SSR 2001	2	58.4	(GTC)9	55	209	AQ326476	[18]
RM27	CORNELL SSR 2001	2	66	(GA)7	55	158	AF343844	[19]
RM561	CORNELL SSR 2001	2	74.1	(GA)11	55	190	AQ913300	[18]
RM6843	IRMI 2003	2	88.2	(TCT)25	55	201	AY023517	[23]
RM5363	IRMI 2003	2	91.8	(TC)13	55	143	AY022038	[23]
RM526	IRMI 2003	2	109.3	(TAAT)5	55	240	AQ794492	[18]
RM6	IRMI 2003	2	125.6	(AG)16	55	163	AF344008	[20]
RM530	IRMI 2003	2	130.2	(GA)23	55	161	AQ840710	[18]
RM213	IRMI 2003	2	150.5	(CT)17	55	139	AF344034	[19]
RM208	IRMI 2003	2	154.1	(CT)17	55	173	AF344029	[19]

Marker	Map	Chr	Position cM	Motif	Annealing Tm (ºC)	Product Size (bp)	GenBank Accession	References
RM60	CORNELL SSR 2001	3	0	(AATT)5AATC T(AATT)	55	165	AF343863	[19]
RM569	IRMI 2003	3	9.3	(GA)30	55	226	AQ864658	[18]
RM545	IRMI 2003	3	24.7	(CT)16	55	175	AQ916814	[18]
RM517	CORNELL SSR 2001	3	42.9	(CT)15	55	266	AQ689735	[18]
RM7	CORNELL SSR 2001	3	64	(GA)19	55	180	AF344009	[20]
RM487	IRMI 2003	3	87.9	(AC)10	55	176	AQ395854	[18]
RM473	CORNELL SSR 2001	3	113.2	(TCTA)14	55	97	AQ331312	[18]
RM426	IRMI 2003	3	122.3	(CA)10	55	150	AQ259184	[18]
RM168	IRMI 2003	3	122.8	T15(GT)14	55	116		[24]
RM186	IRMI 2003	3	127.4	(CGG)5	61	124	D39420	[21]
RM148	CORNELL SSR 2001	3	140.7	(TG)12	55	129		[24]
RM143	CORNELL SSR 2001	3	207.3	(CGG)7	67	207	D78609	[21]
RM130	CORNELL SSR 2001	3	208.2	(GA)10	55	85	D40295	[21]
RM85	CORNELL SSR 2001	3	231	(TGG)5(TCT)12	55	107	AF344087	[21]
RM537	IRMI 2003	4	3.1	(CCG)9	55	236	AQ860402	[18]
RM518	IRMI 2003	4	7.9	(TC)15	55	171	AQ690034	[18]
RM551	CORNELL SSR 2001	4	8.1	(AG)18	55	192	AQ869594	[18]
RM261	CORNELL SSR 2001	4	35.4	C9(CT)8	55	125	AF344080	[19]
RM185	CORNELL SSR 2001	4	50.8	(AGG)9	61	197	D24415	[21]
RM119	IRMI 2003	4	62.6	(GTC)6	67	166		[21]
RM241	IRMI 2003	4	83	(CT)31	55	138	AF344061	[19]
RM3092	IRMI 2003	4	85.5	(AT)49	55	181	AY019767	[23]
RM317	IRMI 2003	4	96	(GC)4(GT)18	55	155	AF344142	[21]
RM159	IRMI 2003	5	5.4	(GA)19	55	248	D48905	[21]
RM122	CORNELL SSR 2001	5	10.1	(GA)7A(GA)2A (GA)11	55	227		[24]
RM413	CORNELL SSR 2001	5	26.7	(AG)11	55	79	AQ157494	[18]

Marker	Map	Chr	Position cM	Motif	Annealing Tm (ºC)	Product Size (bp)	GenBank Accession	References
RM249	CORNELL SSR 2001	5	55.6	(AG)5A2(AG)14	55	121	AF344069	[19]
RM459	IRMI 2003	5	77.4	(CATC)6	55	63	AQ327864	[18]
RM188	IRMI 2003	5	95.3	(CA)8	61	210	D22459	[21]
RM1187	IRMI 2003	5	96.1	(AG)14	50	131	AY017862	[23]
RM421	IRMI 2003	5	101.5	(AGAT)6	55	234	AQ255791	[18]
RM178	IRMI 2003	5	104.4	(GA)5(AG)8	67	117	D49132	[21]
RM26	IRMI 2003	5	111.3	(GA)15	55	112	AF343843	[19]
RM540	CORNELL SSR 2001	6	0	(AG)16	55	172	AQ861615	[18]
RM204	IRMI 2003	6	12.9	(CT)44	55	169	AB026295 .001	[19]
RM225	CORNELL SSR 2001	6	26.2	(CT)18	55	140	AF344046	[19]
RM253	CORNELL SSR 2001	6	37	(GA)25	55	135		[18]
RM549	IRMI 2003	6	51.3	(CCG)9	55	148	AQ868278	[18]
RM1150	IRMI 2003	6	121.7	(AG)13	55	147	AY017825	[23]
RM494	IRMI 2003	6	124.4	(AGA)16	55	203	AQ573378	[18]
RM427	IRMI 2003	7	11	(TG)11	55	185	AQ271555	[18]
RM2	CORNELL SSR 2001	7	36.1	(GA)2A(GA)13	55	150	AF344004	[20]
RM214	IRMI 2003	7	49.7	(CT)14	55	112	APO04305 .001	[19]
RM336	CORNELL SSR 2001	7	61	(CTT)18	55	154	AF344160	[21]
RM182	CORNELL SSR 2001	7	61	(AT)16	67	346	L10346	[22]
RM10	CORNELL SSR 2001	7	63.5	(GA)15	55	159	AF344012	[20]
RM1279	IRMI 2003	7	75.6	(AG)17	50	182	AY017954	[23]
RM3799	IRMI 2003	7	75.9	(GA)19	55	189	AY020474	[23]
RM234	IRMI 2003	7	93.9	(CT)25	55	156	AF344054	[19]
RM1330	IRMI 2003	7	96.1	(AG)20	55	161	AY018005	[23]
RM5720	IRMI 2003	7	115.5	(AAT)22	55	196	AY022395	[23]
RM248	IRMI 2003	7	118	(CT)25	55	102	AF344068	[19]

Marker	Map	Chr	Position cM	Motif	Annealing Tm (ºC)	Product Size (bp)	GenBank Accession	References
RM408	IRMI 2003	8	0.5	(CT)13	55	128	AQ163658	[18]
RM152	IRMI 2003	8	3.3	(GGC)10	55	151	D22858	[22]
RM38	IRMI 2003	8	16.4	(GA)16	55	250	AF343851	[19]
RM6208	IRMI 2003	8	42.9	(CGG)8	55	143	AY022883	[23]
RM210	IRMI 2003	8	53.7	(CT)23	55	140	AF344031	[19]
RM331	IRMI 2003	8	54.3	[(CTT)4GTT]2 (CTT)11	55	176	AF344155	[21]
RM547	CORNELL SSR 2001	8	58.1	(ATT)19	55	235	AQ866086	[18]
RM137	CORNELL SSR 2001	8	60.9	(CT)7	55	218	D47598	[21]
RM80	CORNELL SSR 2001	8	103.7	(TCT)25	55	142	AF343865	[19]
RM308	CORNELL SSR 2001	8	104.8	(AT)4-6-(GT)2T2(GT)7	55	132	AF344133	[21]
RM281	IRMI 2003	8	119.9	(GA)21	55	138	AF344107	[21]
RM219	CORNELL SSR 2001	9	75.6		55			[18]
RM257	CORNELL SSR 2001	9	75.9		55			[18]
RM201	CORNELL SSR 2001	9	93.9	(CT)17	55	158	AF344023	[19]
RM205	IRMI 2003	9	96.1	(CT)25	55	122	AF344026	[19]
RM215	IRMI 2003	9	115.5	(CT)16	55	148	AF344036	[19]
RM245	IRMI 2003	9	118		55			[23]
RM222	IRMI 2003	10	0.5	(CT)18	55	213	AF344043	[19]
RM2504	IRMI 2003	10	3.3	(AT)28	55	184	AY019179	[23]
RM5348	IRMI 2003	10	16.4	(TC)13	55	183	AY022023	[23]
RM216	IRMI 2003	10	42.9	(CT)18	55	146	AF344037	[19]
RM171	IRMI 2003	10	48.8	(GATG)5	55	328	D84275	[22]
RM229	CORNELL SSR 2001	10	58.1	(TC)11(CT)5C3 (CT)5	55	116	AF344049	[19]
RM457	IRMI 2003	11	52.2	(TTAA)5	55	228	AQ327602	[18]
RM21	IRMI 2003	11	53.7	(GA)18	55	157	AF344021	[20]

Table S7. Correlation of SSR markers to trait means for Vandana, Moroberekan and intermated BC_3F_4 lines screened for resistance to rice blast at multi-location trials in India and the Philippines.

SSR Marker	Chr	cM	Trait[1,2]										
			India									Philippines	
			Almora						Hazaribag		Ambikapur	IRRI	Cavinti
			LN	SB	DLA	SD	PB	YB	LB	PB	LB	SB	SB
RM109	2	0	-	-	-	-	0.30[b]	-0.34[b]	0.17	-0.13	0.06	0.52[d]	0.50[d]
RM154	2	4.8	-	-	-	-0.26[a]	-	-	-	-	-	-	-
RM452	2	58.4	0.36[c]	0.42[c]	0.31[b]	-0.26[a]	0.46[d]	-0.46[c]	-	-	-	0.34[c]	0.34[b]
RM545	3	24.7	0.26[a]	-	-	-	-	-	-0.25[a]	-	-	0.28[a]	-
RM517	3	42.9	-	-	0.26[a]	-	0.38[b]	-0.28[a]	-	-	-	-	0.25[a]
RM473D	3	113.2	-0.35[b]	0.32[b]	-0.29[b]	0.37[c]	-0.53[d]	0.55d	-	-	-	-0.32[b]	-0.31[b]
RM426	3	122.3	-	0.32[b]	-	-	0.26[a]	-0.28[a]	-	-0.26[a]	-	0.32[b]	0.31[b]
RM168	3	122.8	0.34[c]	-	0.36[c]	-0.31[b]	0.52[d]	-0.44[c]	-	-	-	0.25[a]	0.25[a]
RM186	3	127.4	0.26[a]	-	0.25[a]	-0.29[a]	0.25[a]	-0.38[b]	0.35[c]	-	0.30[b]	0.26[a]	0.33[b]
RM143	3	207.3	-0.28[a]	0.25[a]	-0.33[a]	-	-	0.26[a]	-	-	-	-	-
RM85	3	231	0.29[a]	-	-	-0.25a	0.37b	-0.32[b]	-	-	-	0.29a	0.30b
RM413	5	26.7	-	-	-	-	-	-	-	-	-	-0.25[a]	-
RM421	5	101.5	0.30[a]	-	0.28[a]	-0.27[a]	0.41[c]	-0.33[b]	-	-	-	-	-
RM178	5	104.4	-	-	-	-	0.33b	-0.34[b]	-	-	-	-	-
RM26	5	111.3	-	-	0.33[b]	-	-	-	-	-	-	-	-
RM2	7	36.1	0.33[b]	0.35[c]	0.26[a-]	0.39[c]	0.46[c]	-0.43[c]	-	-	-	0.39[b]	0.37[b]
RM1330	7	96.1	-	-	-	-	-	-	-	0.31[b]	-	-	-
RM408	7	118.0	0.32[b]	-	0.31[b]	-0.29[b]	0.48[d]	-0.52[d]	-	-	-	0.24[a]	0.25[a]
RM152	8	3.3	0.28[a]	0.33[b]	-0.29[b]	-0.29a	0.37b	-0.45[c]	-	-	-	0.37[c]	0.37[c]
RM3215	8	49.5	-	-	-	-	-	-	-	-	-	-	-0.25[a]

Table S7 (continued)

**SSR Marker**	**Chr**	**cM**	**Trait[1,2]**										
			India									Philippines	
			Almora						Hazaribag		Ambikapur	IRRI	Cavinti
			LN	SB	DLA	SD	PB	YB	LB	PB	LB	SB	SB
RM25	8	52.2	-	-	0.25[a]	-0.25[a]	-	-	-	-	-	-	-
RM331	8	54.3	-	-	-	-	-	-	-	-0.55[d]	-	-	-
RM137	8	58.1	-	-	-	-	-	-	-	-	0.35[b]	-	-
RM547	8	58.1	-	-	-	-	-	-0.30[a]	-	-	-	-	-
RM308	8	104.7	-	-	-	-	-	-	-	-0.26[a]	-	0.26[a]	-0.26[a]
RM281	8	119.9	0.27[a]	0.27[a]	0.27[a]	-	0.43[c]	-0.43[c]	-	-	-	0.26[a]	0.27[a]

[1] LN = lesion number; SB = seedling blast; DLA = diseased leaf area; SD = start of disease (days after sowing); PB = panicle blast; YB = yield under blast condition; LB = leaf blast

[2] Significance of correlation is: a = $p < 0.05$; b = $p < 0.01$; c = $p < 0.001$; d = $p < 0.0001$

Table S8. SSR markers correlated with resistance to rice blast in India and the Philippines that co-localize with known QTL for resistance to biotic stress[1].

Marker	Chr	cM	BB	RB	BPH	SB	References
RM109	2	0	x	x			1,25
RM154	2	4.8	x	x			1
RM452	2	58.4				x	26
RM545	3	24.7		x	x		1,26 2
RM517	3	42.9		x	x		2,26
RM473D	3	113.2					1
RM143	3	207.3				x	27
RM85	3	231					1
RM413	5	26.7		x			1,28
RM421	5	101.5		x		x	25,29
RM178	5	104.4				x	29
RM2	7	36.1		x			25,30
RM408	7	118.0		x		x	27,30
RM152	8	3.3		x		x	28,30,31
RM3215	8	49.5		x	x	x	28,30,31
RM25	8	52.2	x	x	x	x	1,28,30-32
RM331	8	54.3	x	x		x	1,28,30-32
RM137	8	58.1	x	x		x	1,28,30-32
RM547	8	58.1	x	x		x	1,28,30,32
RM308	8	104.7			x		33
RM281	8	119.9	x		x		1,26,32

[1] BB = bacterial blight; RB = rice blast; BPH = brown plant hopper; SB = sheath blight

Table S9. Field performance of Vandana, Moroberekan and selected intermated BC_3F_4 lines screened for resistance to rice blast at multi-location trials in India and the Philippines.

Line	Trait/Location										
	India									Philippines	
	Almora						Hazaribag		Ambikapur	IRRI	Cavinti
	LN	SB	DLA	SD	PB	YB	LB	PB	LB	SB	SB
Moroberekan	0.70	3.5	1.5	20	3.2	-	0	0	1.5	1.0	1.0
IR78221-19-6-7-B-B	1.1	2.5	1.0	25	3.3	15.35	2.0	20.8	4.0	1.0	1.0
IR78221-19-6-33-B-B	1.4	5.0	2.0	15	3.9	105.0	2.0	2.4	4.0	1.0	1.0
IR78221-19-4-54-B-B	1.2	4.5	1.5	15	3.5	152.5	2.0	51.3	2.0	1.0	1.0
IR78221-19-6-56-B-B	0.9	4.0	1.0	25	3.1	80.0	2.0	1.3	2.5	1.0	1.0
IR78221-19-6-82-B-B	0.5	3.0	1.0	20	3.4	165.0	2.0	7.5	1.0	1.0	1.0
IR78221-19-6-90-B-B	0.3	6.0	1.0	20	3.0	112.5	3.0	5.0	2.5	1.0	1.0
IR78221-19-6-99-B-B	0.9	6.0	1.0	25	3.3	170.5	2.0	10.2	4.0	1.0	1.0
IR78222-20-7-148-2-B	1.2	5.0	1.5	20	5.1	70.5	2.0	3.0	4.0	4.0	4.0
IR78221-19-3-196	2.4	2.5	57.5	15	8.3	5.0	3.0	10.0	4.0	4.0	4.0
Vandana	3.3	8.5	65.0	15	8.3	5.0	4.0	10.0	5.0	7.0	8.0

LN = lesion number; SB = seedling blast (Scale 0-9); DLA = % diseased leaf area; SD = start of disease (days after sowing); PB = panicle blast (Scale 0-9); YB = yield under blast condition (grams/plot); LB = Leaf blast

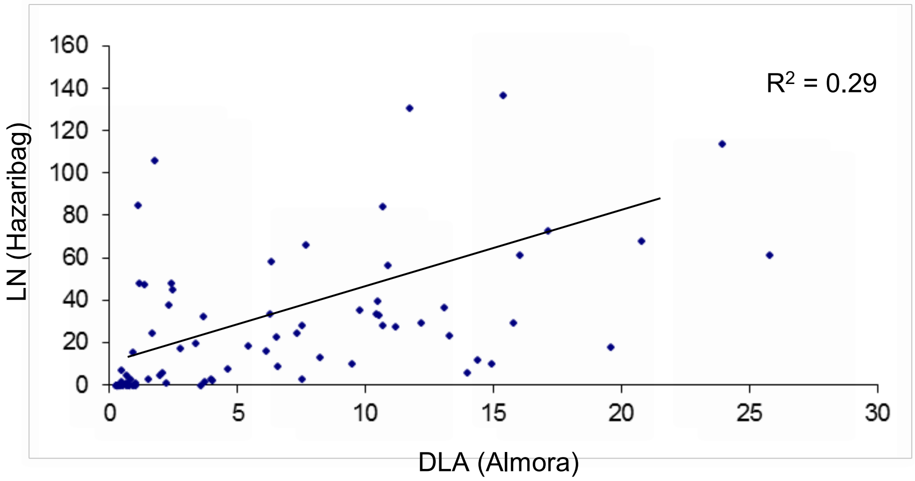

Fig. S1. Correlation of disease leaf area at Almora and lesion number at Hazaribag, India on F_4 lines from intermated BC_3F_4 families. Measurements showed a positive correlation (r= 0.54**, n=80 2005). LN = Lesion number, DLA = Disease leaf area.

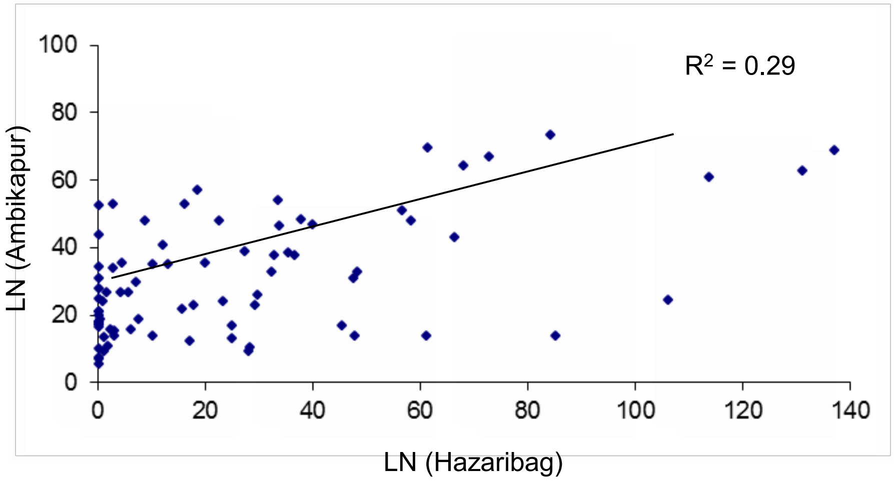

Figure S2. Correlation of Lesion Number (LN) in Ambikapur and lesion number in Hazaribag, India on F_4 lines from intermated BC_3F_4 families. LN counts at both sites showed a significantly positive correlation (r= 0.546**, n=80 2005).

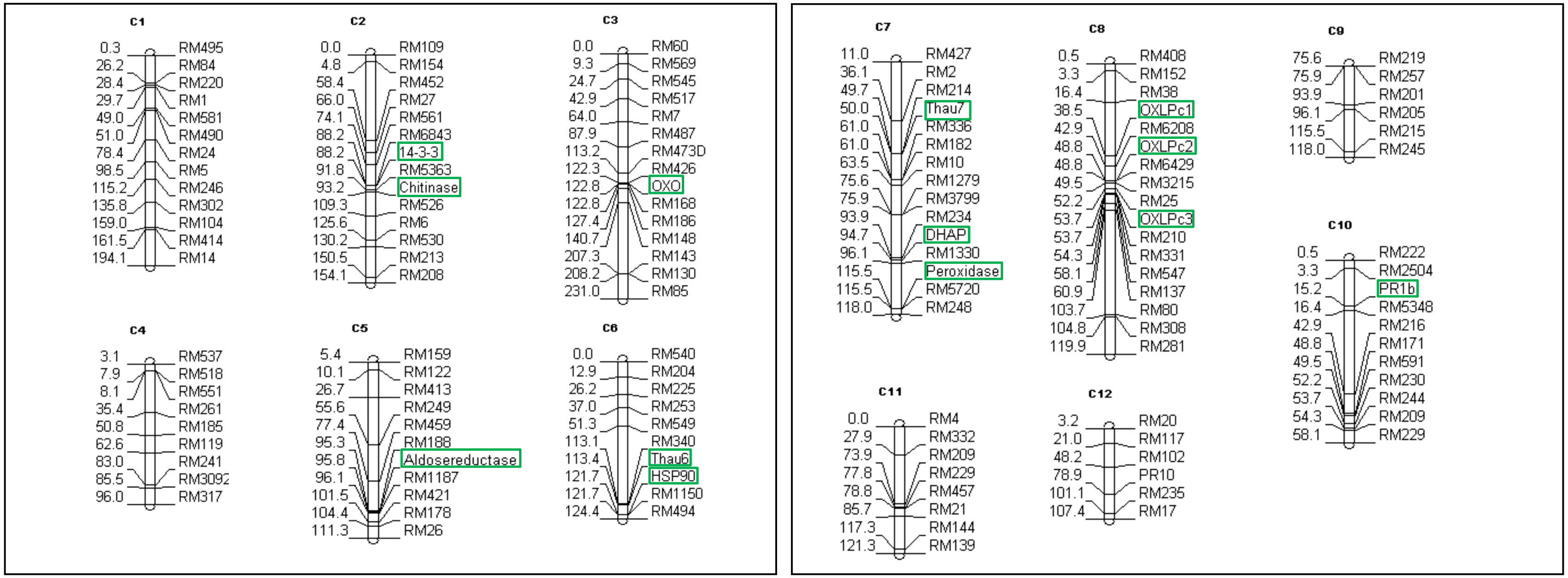

Figure S3. Location of SSR markers and candidate genes (green rectangles) considered in the study. OXLP = GLP.

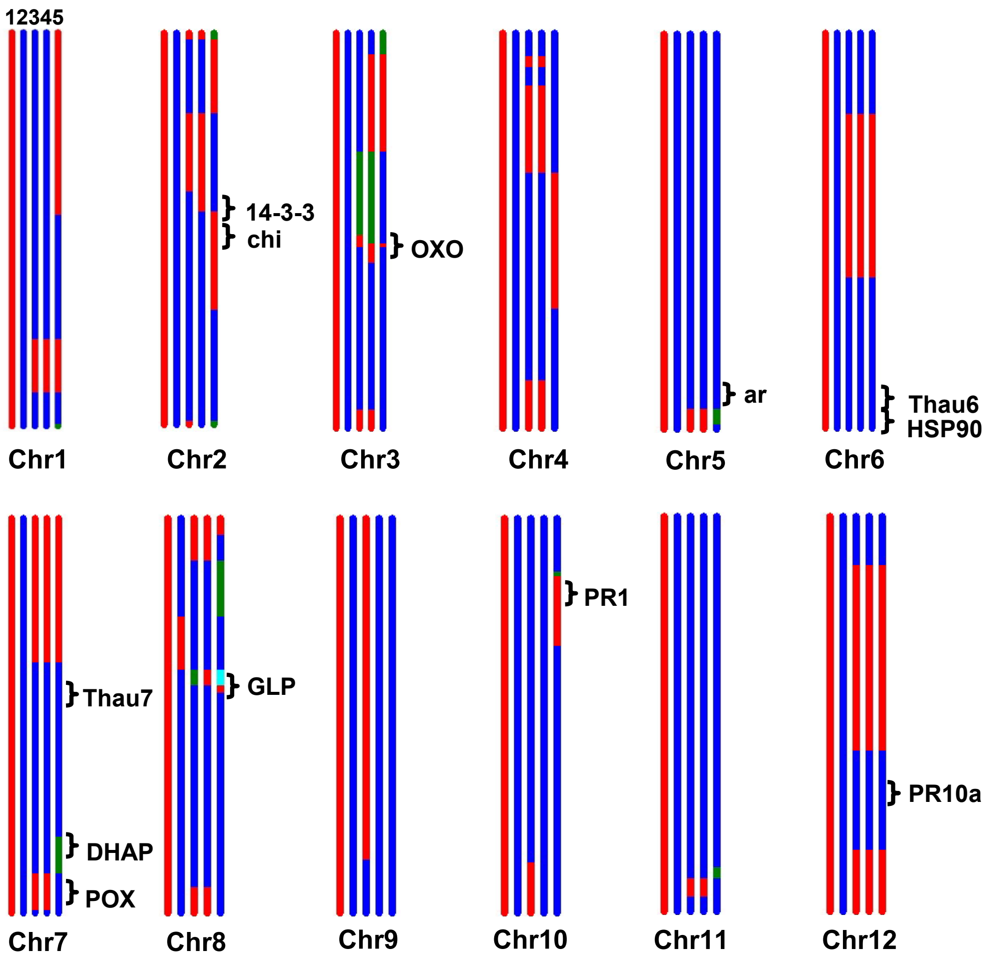

Figure S4. Diagram representing data from genome-wide scan of parental lines and a selected subset of F_6 intermated lines. Moroberekan (1- red), and Vandana (2 – blue) genomic regions are shown in three selected lines IR78221-19-6-33-B (3), IR78221-19-6-90-B (4), IR78222-20-7-148-2-B (5) all of which show Resistant phenotype. While most of the genomic background is similar to Vandana, some regions, including QTLs containing CGs, are from Moroberekan. Green regions represent heterozygosity.

Literature Cited in the Supplemental Documents

1 Ramalingam, J. *et al.* Candidate defense genes from rice, barley, and maize and their association with qualitative and quantitative resistance in rice. *Mol Plant Microbe Interact* **16**, 14-24 (2003).

2 Chen, F., Li, Q., Sun, L. & He, Z. The rice 14-3-3 gene family and its Involvement in responses to biotic and abiotic stress. *DNA Res* **13**, 53 (2006).

3 Karuna Sree, B., Rajendrakumar Chadalavada, S. V. & Reddy, A. R. Aldose redutase in rice (*Oryza sativa* L.): stress response and developmental specificity. *Plant Sci.* **160**, 149-157 (2009).

4 Bishop, J. G., Dean, A. M. & Mitchell-Olds, T. Rapid evolution in plant chitinases: molecular targets of selection in plant-pathogen coevolution. *Proc Natl Acad Sci U S A* **97**, 5322-5327 (2000).

5 Takahashi, A. *et al.* Hyperphosphorylation of a mitochondrial protein, prohibitin, is induced by calyculin A in a rice lesion-mimic mutant cdr1. *Plant Physiol* **132**, 1861-1869, doi:10.1104/pp.103.021733 (2003).

6 de la Fuente, S. *et al.* Heat shock protein 90 and its co-chaperone protein phosphatase 5 interact with distinct regions of the tomato I-2 disease resistance protein. *Plant J* **43**, 284-298, doi:10.1111/j.1365-313X.2005.02450.x (2005).

7 Faris, J. D., Li, W. L., Liu, D. J., Chen, G. D. & Gill, b. S. Candidate gene analysis of quantitative disease resistance in wheat. *Theor Appl Genet* **V98**, 219 (1999).

8 Liu, B. *et al.* Candidate defense genes as predictors of quantitative blast resistance in rice. *Mol Plant Microbe Interact* **17**, 1146-1152 (2004).

9 Wu, J. L. *et al.* Association between molecular markers and blast resistance in an advanced backcross population of rice. *Theor Appl Genet* **108**, 1024-1032 (2004).

10 Carrillo, M. G. C., Goodwin, P. H., Leach, J. E., Leung, H. & Vera Cruz, C. M. Phylogenomic relationships of rice oxalate oxidases to the cupin superfamily and their association with disease resistance QTL. *Rice* **2**, 67-79, doi:10.1007/s12284-009-9024-0 (2009).

11 Davidson, R. M. *et al.* Rice germin-like proteins: Allelic diversity and relationships to early stress responses. *Rice* **3**, 43-55, doi:10.1007/s12284-010-9038-7 (2010).

12 Manosalva, P. M. *et al.* A germin-like protein gene family functions as a complex quantitative trait locus conferring broad-spectrum disease resistance in rice. *Plant Physiol* **149**, 286-296, doi:10.1104/pp.108.128348 (2009).

13 Zheng, B. S. *et al.* Mapping QTLs and candidate genes for rice root traits under different water-supply conditions and comparative analysis across three populations. *Theor Appl Genet* **107**, 1505-1515 (2003).

14 Trudel, J., Grenier, J., Potvin, C. & Asselin, A. Several thaumatin-like proteins bind to beta-1,3-glucans. *Plant Physiol* **118**, 1431-1438 (1998).

15 Woloshuk, C. P., Meulenhoff, J. S., Sela-Buurlage, M., van den Elzen, P. J. & Cornelissen, B. J. Pathogen-induced proteins with inhibitory activity toward Phytophthora infestans. *Plant Cell* **3**, 619-628, doi:10.1105/tpc.3.6.619 (1991).

16 Chittoor, J. M., Leach, J. E. & White, F. F. Differential induction of a peroxidase gene family during infection of rice by *Xanthomonas oryzae* pv. *oryzae*. *Mol Plant Microbe Interact* **10**, 861-871 (1997).

17 Midoh, N. & Iwata, M. Cloning and characterization of a probenazole-inducible gene for an intracellular pathogenesis-related protein in rice. *Plant Cell Physiol* **37**, 9-18, doi:10.1093/oxfordjournals.pcp.a028918 (1996).

18 Temnykh, S. *et al.* Computational and experimental analysis of microsatellites in rice (*Oryza sativa* L.): frequency, length variation, transposon associations, and genetic marker potential. *Genome Res* **11**, 1441-1452, doi:10.1101/gr.184001 (2001).

19 Chen, X., Temnykh, S., Xu, Y., Cho, Y. G. & McCouch, S. R. Development of a microsatellite framework map providing genome-wide coverage in rice (*Oryza sativa* L.). *Theor Appl Genet* **95**, 553-567, doi:DOI 10.1007/s001220050596 (1997).

20 Panaud, O., Chen, X. & McCouch, S. R. Development of microsatellite markers and characterization of simple sequence length polymorphism (SSLP) in rice (*Oryza sativa* L.). *Mol Gen Genet* **252**, 597-607, doi:10.1007/bf02172406 (1996).

21 Temnykh, S. *et al.* Mapping and genome organization of microsatellite sequences in rice (*Oryza sativa* L.). *Theor Appl Genet* **100**, 697-712, doi:DOI 10.1007/s001220051342 (2000).

22 Akagi, H., Yokozeki, Y., Inagaki, A. & Fujimura, T. Microsatellite DNA markers for rice chromosomes. *Theor Appl Genet* **93**, 1071-1077, doi:10.1007/BF00230127 (1996).

23 McCouch, S. R. *et al.* Development and mapping of 2240 new SSR markers for rice (*Oryza sativa* L.) *DNA Res* **9**, 257-279 (2002).

24 Wu, K. S. & Tanksley, S. D. Abundance, polymorphism and genetic mapping of microsatellites in rice. *Mol Gen Genet* **241**, 225-235, doi:10.1007/bf00280220 (1993).

25 Eizenga, G. C., Agrama, H. A., Lee, F. N., Yan, W. & Jia, Y. Identifying novel resistance genes in newly introduced blast resistant rice germplasm. *Crop Sci* **46**, 1870-1878, doi:10.2135/cropsci2006.0143 (2006).

26 Alam, S. N. & Cohen, M. B. Detection and analysis of QTLs for resistance to the brown planthopper, *Nilaparvata lugens*, in a doubled-haploid rice population. *Theor Appl Genet* **97**, 1370-1379, doi:DOI 10.1007/s001220051031 (1998).

27 Kunihiro, Y. *et al.* QTL analysis of sheath blight resistance in rice (*Oryza sativa* L.). *Yi Chuan Xue Bao* **29**, 50-55 (2002).

28 Wang, G. L. *et al.* RFLP mapping of genes conferring complete and partial resistance to blast in a durably resistant rice cultivar. *Genetics* **136**, 1421-1434 (1994).

29 Han, Y. P. *et al.* Mapping QTLs for horizontal resistance to sheath blight in an elite rice restorer line, Minghui 63. *Yi Chuan Xue Bao* **29**, 622-626 (2002).

30 Xing, Z. *et al.* Characterization of the main effects, epistatic effects and their environmental interactions of QTLs on the genetic basis of yield traits in rice. *Theor Appl Genet* **105**, 248-257, doi:10.1007/s00122-002-0952-y (2002).

31 Li, Z., Pinson, S. R., Marchetti, M. A., Stansel, J. W. & Park, W. D. Characterization of quantitative trait loci (QTLs) in cultivated rice contributing to field resistance to sheath blight (*Rhizoctonia solani*). *Theor Appl Genet* **91**, 382-388, doi:10.1007/BF00220903 (1995).

32 Li, Z. K. *et al.* Are the dominant and recessive plant disease resistance genes similar? A case study of rice R genes and *Xanthomonas oryzae* pv. *oryzae* races. *Genetics* **159**, 757-765 (2001).

33 Xu, X. F., Mei, H. W., Luo, L. J., Cheng, X. N. & Li, Z. K. RFLP-facilitated investigation of the quantitative resistance of rice to brown planthopper (*Nilaparvata lugens*). *Theor Appl Genet* **104**, 248-253, doi:10.1007/s00122-001-0777-0 (2002).
